# Cerebellar and premotor activity during a non-fatiguing grip task reflects motor fatigue in relapsing-remitting multiple sclerosis

**DOI:** 10.1371/journal.pone.0201162

**Published:** 2018-10-24

**Authors:** Olivia Svolgaard, Kasper Winther Andersen, Christian Bauer, Kristoffer Hougaard Madsen, Morten Blinkenberg, Finn Selleberg, Hartwig Roman Siebner

**Affiliations:** 1 Danish Research Centre for Magnetic Resonance, Centre for Functional and Diagnostic Imaging and Research, Copenhagen University Hospital Hvidovre, Hvidovre, Denmark; 2 Metropolitan University College, Copenhagen, Denmark; 3 Department of Applied Mathematics and Computer Science, Technical University of Denmark, Kongens Lyngby, Denmark; 4 Danish Multiple Sclerosis Center, Copenhagen University Hospital Rigshospitalet, Copenhagen, Denmark; 5 Department of Neurology, Copenhagen University Hospital Bispebjerg, Copenhagen, Denmark; Charite Universitatsmedizin Berlin, GERMANY

## Abstract

Fatigue is a common and highly disabling symptom of multiple sclerosis. Patients experience an effort-independent general subjective feeling of fatigue as well as excessive fatigability when engaging in physical or mental activity. Previous research using functional magnetic resonance imaging (fMRI) has revealed heterogeneous findings, but some evidence implicates the motor system. To identify brain correlates of fatigue, 44 mildly impaired patients with relapsing-remitting multiple sclerosis and 25 age- and gender-matched healthy controls underwent functional magnetic resonance imaging at 3 Tesla, while they performed alternating blocks of rest and a non-fatiguing precision grip task. We investigated neural correlates of fatigue using the motor subscore of Fatigue Scale for Motor and Cognitive Functions (FSMC_MOTOR_) using the bilateral motor cerebellum, putamen, and dorsal premotor cortex as regions of interest. Patients and healthy controls performed the grip force task equally well without being fatigued. In patients, task-related activity in lobule VI of right motor cerebellum changed in proportion with individual FSMC_MOTOR_ scores. In right dorsal premotor cortex, linear increases in activity across consecutive task blocks scaled with individual FSMC_MOTOR_ scores in healthy controls, but not in patients. In premotor and dorsomedial prefrontal areas, patients were impaired at upscaling task-related activity the more they were affected by motor fatigue. The results support the notion that increased sensorimotor processing in the cerebellum contributes to the experience of motor fatigue and fatigability in multiple sclerosis. Additionally, downscaling of motivational input or sensorimotor processing in prefrontal and premotor areas may constitute an additional pathophysiological factor.

## Introduction

Fatigue is one of the most common and disabling symptoms in multiple sclerosis (MS) [1–3]. Fatigue is a complex phenomenon to study because the patients experience an effort-independent general subjective feeling of fatigue as well excessive fatigability when engaging in physical or mental activity [4]. Effort-independent fatigue is often referred to as the “trait” fatigue, whereas fatigability is referred to as a “state” feature of fatigue. In a clinical setting, the two aspects are usually measured jointly using standardized scales [4–6].

The precise mechanisms that determine the emergence and magnitude of effort-independent “state” fatigue and effort-evoked “trait” fatigability in a given patient are still largely unknown and treatment remains a challenge [7]. Whatever the causal mechanisms may be, the experience of excessive fatigue and fatigability are underpinned by pathophysiological changes in functional brain networks [8]. Identifying brain activity that scales with the experience of fatigue and fatigability during everyday life may not only reveal important insights into the pathophysiology of fatigue, but may be a step towards establishing neuroimaging-based biomarkers of fatigue that can supplement the subjective clinical scores.

Functional magnetic resonance imaging (fMRI) has been used in patients with MS to link task-related brain activity patterns to subjectively experienced fatigue during everyday life, yet results have been relatively incongruent across studies [8–13]. This can be attributed to the fact that MS is a heterogeneous disease and most studies examined relatively few patients. Additionally, there are several methodological differences in the studies, e.g. regarding the experimental tasks, the MS phenotypes, age range, disease-related disability (i.e. as reflected by EDSS scores), disease durations, fatigue questionnaires, etc. Only a couple of the studies share some common ground regarding the fatigue related brain activation changes [8, 9, 13, 14]. Specogna et al. [13] acquired fMRI, while patients performed a self-paced, sequential, thumb-to-finger opposition task. Fatigue was assessed using the Fatigue Severity Scale (FSS) [15]. In fatigued patients with a score above 5, task-induced fatigue was associated with stronger task-related activity in right middle frontal gyrus, dorsal premotor cortex and putamen compared to non-fatigued patients with a score below 4 [13]. Pardini et al. [9] studied mildly affected patients, while they performed an acoustically paced, sequential thumb-to-finger opposition task twice per second. None of the patients perceived the task as fatiguing. Using the Modified Fatigue Impact Scale as index of fatigue (MFIS) during everyday life [16], a positive correlation was found between experienced fatigue and task-related activity in right motor cerebellum (lobule VI) as well as orbitofrontal cortex [9]. In another fMRI study, mildly disabled patients and healthy controls made repetitive finger flexion–extension movements [8]. Healthy controls showed a linear increase in task-related activity across task blocks in right and left putamen, and left precentral cortex [8]. This time-dependent linear increase in activity was reduced in patients who suffered from fatigue [8]. In a recent fMRI study, 14 MS patients performed an acoustically paced, sequential thumb-to-finger opposition task with their right hand twice per second to test fatigability by evoking effort-induced fatigue [14]. Among other parts of the basal ganglia, patients recruited the putamen already at the beginning of the task before fatigability arose [14]. This initial activation differs from the activity pattern found in healthy subjects [17]. Healthy subjects recruited the putamen first after continuous task performance had induced a state of fatigue, but not at the beginning of task performance [17]. Furthermore, other fMRI tasks have been used to study neural correlates of fatigue in MS such as stress or reward-processing tasks [18, 19].

This current study took a new step in the search of the pathophysiological changes in functional brain networks related to fatigue in a well-defined group of MS patients. Extending previous fMRI studies, we chose a tonic precision grip task as sensorimotor paradigm, because this task required the continuous integration of visual and somatosensory input with the generated motor output to maintain the required target force level [20]. We reasoned that the nature of the task would engage sensorimotor control regions that contribute to motor fatigue in MS. We performed whole-brain fMRI to further clarify the relation between sensorimotor brain activity during a non-fatiguing sensorimotor task and the amount of motor fatigue and fatigability experienced during everyday life. Since previous fMRI studies of repetitive finger movements pointed to an alteration of task-related activity in dorsal premotor cortex (PMd), motor cerebellum, and putamen [8, 9, 13, 14], we defined these areas as region of interest. We expected that patients suffering from motor fatigue and fatigability in their everyday life would display overall differences in task-related recruitment or differences in the time-dependent modulation of task-related activity levels in these areas. We further hypothesized that task-related activity in the ROIs would reflect the amount of motor fatigue and fatigability that patients experience during daily activities.

## Material and methods

### Participants

50 patients with relapsing-remitting MS and 25 healthy individuals were initially included in the study. Inclusion criteria were age between 18 to 55 years and right-handedness according to Edinburgh Handedness Inventory [21]. Patients had to have a maximal Expanded Disability Status Scale (EDSS) score of 3.5 and to be attack-free and on same treatment for the last three months. Exclusion criteria were pregnancy, contraindication for MRI, pharmacological treatment of fatigue, medical or psychiatric comorbidity, history of infection, sleeping problems, drug, or alcohol abuse. Approval was given by the Ethics committee of the Capital Region of Denmark (Protocol H-4-2013-182) and written informed consent was obtained before inclusion in the study.

### Clinical assessment

Self-reported fatigue was assessed with the Danish validated version of the Fatigue Scale for Motor and Cognitive Functions (FSMC) [22, 23]. The questionnaire has been developed to assess the subjective experience of fatigue in MS patients during normal day-to-day life in general. The questions capture both, effort-independent “trait” fatigue and effort-dependent “state” fatigue (i.e., fatigability). The questionnaire has a motor subscale and a cognitive subscale and gives three measures of fatigue: an overall score, a motor score and a cognitive score ranging from 20–100 and 10–50, respectively. There is a high correlation between the cognitive and motor sub-scale of the questionnaire [23]. Since the focus of this study was on the motor component of fatigue and fatigability, only the motor sub-score of the Fatigue Scale for Motor and Cognitive Functions (FSMC_MOTOR_) was considered and used to quantify the magnitude of motor fatigue and fatigability experienced during everyday life. Based on the individual FSMC_MOTOR_ score, patients were divided into patients with fatigue (score ≥ 27) and patients without fatigue (FSMC_MOTOR_ score ≥ 27) and patients without fatigue (score ≥ 27) and patients without fatigue (FSMC_MOTOR_ score < 27) corresponding to the cut-off point for moderate motor fatigue [22]. Overall disease-related disability was assessed with the EDSS [24]. Skilled hand function was quantified with the Nine Hole Peg Test (9-HPT) [25] and Jebsen-Taylor Hand Function Test (JTHFT) [26]. The severity of depressive symptoms, cognitive impairment, sleep problems were assessed with well-established tests or questionnaires such as the Beck Depression Inventory II (BDI-II) [27, 28], Symbol Digit Modality Test (SDMT) [29], Paced Auditory Serial Addition Test (PASAT) [30], Epworth Sleepiness Scale (ESS) [31, 32], and Pittsburgh Quality of Sleep Index (PQSI) [33].

### Magnetic resonance imaging

Whole-brain MRI was performed with an Achieva 3 Tesla scanner and a 32-channel head coil (Philips, Best, The Netherlands). Blood oxygen level dependent fMRI [34] was acquired using Echo Planar Imaging (EPI) with a repetition time (TR) of 2500 ms, echo time (TE) of 30 ms and flip-angle of 80°. Each brain volume consisted of 42 axial slices acquired in interleaved order with a slice-thickness of 3 mm and 3x3x3 mm voxel resolution, covering a field-of-view (FOV) of 192x192x126 mm. Whole-brain fMRI was acquired throughout three phases, a pre-fatigue phase, a fatigue induction phase, and a post-fatigue induction phase. This paper is only using the fMRI data, which were acquired in the pre-fatigue phase, corresponding to the first 201 acquired brain volumes of the fMRI session. The fatigue induction phase was variable across subjects due to the individual differences in fatigability and lasted from 39 seconds to 494 seconds and was used to induce motor fatigue in the subjects. The post-fatigue phase was similar to the pre-fatigue phase, so the total duration of the fMRI scan was between 19.3 minutes and 28.5 minutes (including pauses in between the different phases). Here we will only report the results from the first pre-fatigue phase, results for the two other phases will be presented elsewhere.

Structural MRI scans were additionally acquired to measure lesion load and brain atrophy and included a three-dimensional high-resolution T1-weighted image acquired with a sagittal magnetization prepared rapid acquisition gradient echo (MPRAGE) sequence (TR = 6 ms, TE = 2.70 ms; flip-angle = 8°, 0.85 mm isotropic voxel size, FOV = 245x245x208 mm). A T2-weighted image was acquired with a turbo spin echo sequence (TR = 2500 ms, TE = 270 ms; flip-angle = 90°, 0.85 mm isotropic voxel size, FOV = 245x245x190 mm) and a fluid attenuated inversion recovery image (FLAIR) (TR = 4800 ms, TE = 327ms, 1 mm isotropic voxel size, FOV = 256x256x202 mm). We also performed diffusion weighted MRI of the brain, which will be reported separately.

### Tonic grip force task during fMRI

**Fig 1:**
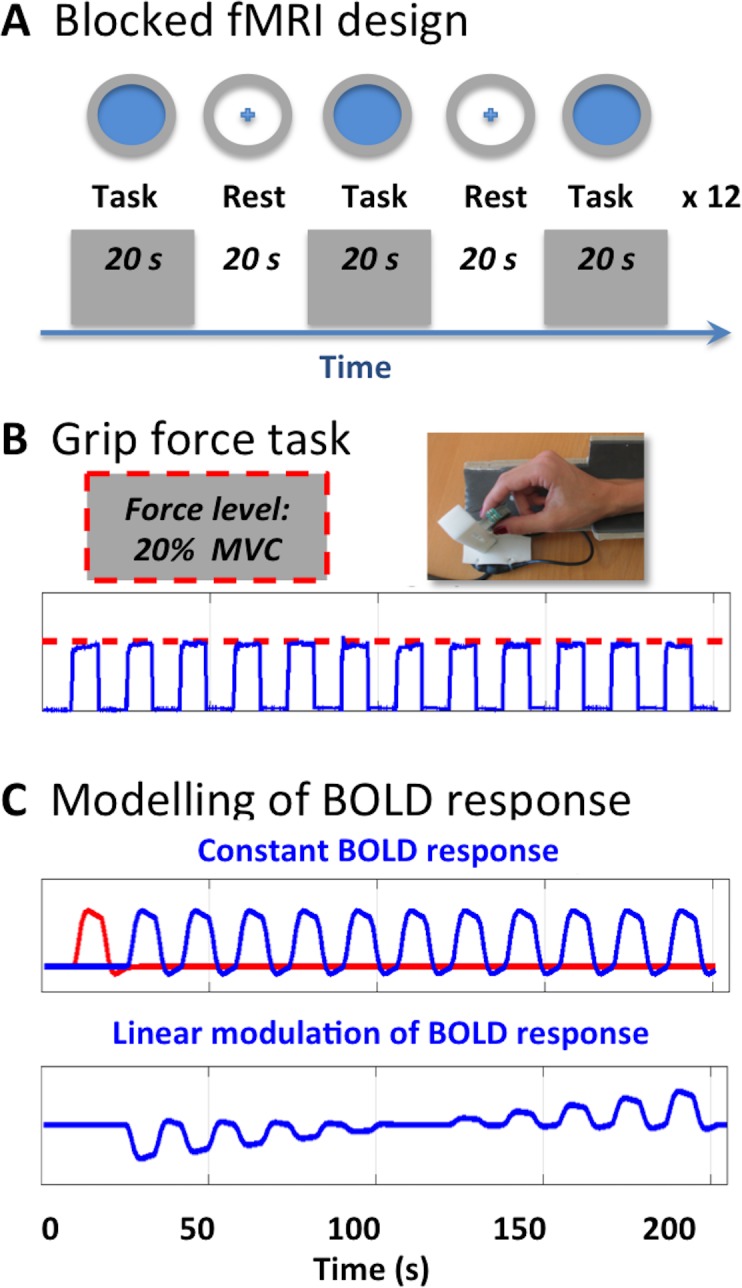
The precision grip task and parametric modulation. **(A)** The tonic precision grip task consisted of 12 task blocks (each 20 s) alternated by periods of rest (20 s). During the grip task, visual feedback of the exerted grip force (blue area) and the required target force (grey circle) was continuously provided. **(B)** Participants had to maintain a target force corresponding to 20% of the individual maximal voluntary contraction and the grip force output was continuously recorded. **(C)** Task-related activity was analysed using a General Linear Model in which the constant main effect of task (main effect) as well as linear modulation of task-related activity (linear time effect) were modelled. Task-related activation during the first block (red line) was separately modelled and treated as effect of no interest.

Detailed oral and written task instructions were given and the subjects were familiarized with the task before fMRI. Participants were holding a force sensitive device with their right hand using a pincer grip and produced a steady force level, which was individually adjusted to 20% of their maximal force. The force produced by the participant was continuously visualized as expanding circle on a screen. The circle had to match the size of a ring, which indicated the target force level (Fig 1). To avoid fatigue, alternating 20-s task blocks, during which subjects produced a force level at 20% of individual maximal voluntary contraction (MVC), alternated with 20-s periods of rest. Performance was continuously monitored on a screen in the MRI control room. The acquired grip force data was scaled to reflect force in Newton and the mean and standard deviation of the applied force were extracted using Matlab (The Mathworks Inc., USA, https://se.mathworks.com/products/matlab.html).

### Pre-processing and analysis of MRI data

Lesions were automatically delineated on the FLAIR images using Jim software (JIM version 6.0 Xinapse System, Leicester, UK, http://www.xinapse.com/). The delineated lesions were checked and if necessary, corrected by an expert using the co-registered T2- and T1-weighted images as support. The individual subject’s T1-weighted image was normalized to Montreal Neurological Institute (MNI) space using Statistical Parametric Mapping software (SPM 12, Welcome Department of Imaging Neuroscience, London, UK, http://www.fil.ion.ucl.ac.uk/spm/software/) and the normalization warp was then applied to the lesion mask. The normalized lesion masks were then summed across subjects to form a lesion frequency image in MNI space.

Structural reconstruction and volumetric segmentation of structural MRIs were performed with FreeSurfer software (version 5.3.0; http://surfer.nmr.mgh.harvard.edu) using a standard processing pipeline [35, 36], which includes intensity normalization to MNI space, skull stripping, filtering, segmentation, and surface deformation. To increase the segmentation accuracy for the patients, the semi-automated lesion delineations were entered into FreeSurfer as white matter hypointensity (on T1w) voxels in the segmentation pipeline [37]. Quality of the skull stripping and accuracy of grey and white matter outer boundaries were reviewed by a trained researcher. The volumetric data of estimated total intracranial volume (TIV), white matter volume (WMV), and grey matter volume (GMV) were extracted using specialized FreeSurfer tools for automated parcellation of grey and white matter [38]. The extracted volume measures were transformed into z-scores for further analyses.

The fMRI data were analysed using SPM 12. Images were realigned to the mean EPI image using a six-parameter, rigid-body transformation and spatial normalized to the MNI ICMB European brain template, using the mean realigned image to determine the transformation. The images were resampled to 2x2x2 mm3 voxels in MNI space and smoothed with a 6 mm full-width at half-maximum isotropic Gaussian kernel. For each participant, a general linear model (GLM) was used to model the fMRI time series. The design matrix modelled task-related activation as a boxcar function reflecting the alternation of task and rest periods convolved with the canonical hemodynamic response function. A second regressor modelled the first-order (linear) modulation of task-related BOLD signal changes across the session orthogonalized to the main task regressor [39]. The design matrix included additional 24 nuisance regressors derived from the realignment parameters [40] and a regressor-of-no-interest for the first block of the tonic precision grip task during which subjects reached steady-state performance.

### Statistical analyses

Group analysis of the fMRI data employed random effects analysis to test for voxel-wise significance within and between groups, yielding mean statistical parametric maps for each group and for between-group difference using one-sample and two-sample T-tests, respectively. The group models for within and between group analyses contained the FSMC_MOTOR_ score as effect of interest, and the two nuisance regressors “age” and “hand function” measured with the JTHFT. Separate group models were set up for the main effect of task and the first-order modulation of task-related activation during the session.

Group analyses tested for differences in task-related activity between healthy controls and patients with MS as well as differences between patients with excessive fatigue (FMS group) and patients without fatigue (NFMS group). In the latter analysis, individual BDI-II scores were included as a covariate in the model to isolate the effect of fatigue from those associated with the presence of depressive symptoms.

**Fig 2:**
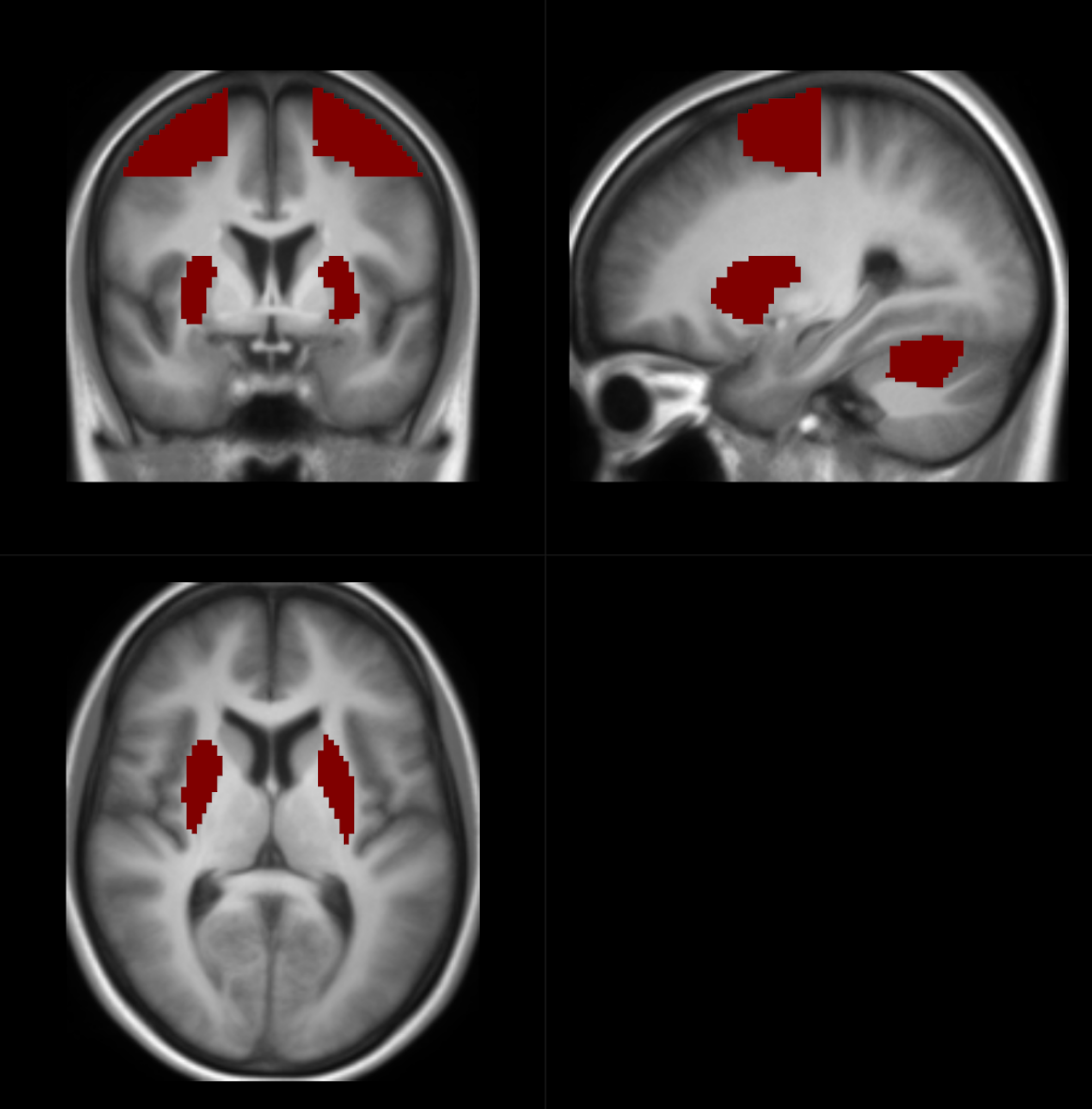
The mask used to define the region of interest. A single mask consisting of the cerebellum lobule VI, putamen, and PMd of both hemispheres were used to define the region of interest.

The correction for multiple non-independent comparisons at the peak voxel level was performed using the family-wise error method implemented in SPM based on Gaussian random fields and the statistical threshold was set to p < 0.05. A single mask consisting of the cerebellum lobule VI, putamen, and PMd of both hemispheres were used to define the region of interest (Fig 2). The PMd ROI was defined based on the Human Motor Area Template (HMAT) number 9 and 10 [41] converted from Talairach to MNI space with tal2mni (http://eeg.sourceforge.net/doc_m2html/bioelectromagnetism/tal2mni.html). The putamen ROI was defined with the Automated Anatomic Labelling Atlas (AAL) [42]. The cerebellar lobule VI was defined with the probabilistic MRI cerebellum atlas by Diedrichsen et al. [43]. For voxels within the ROI, small volume correction was applied considering all voxels within the ROI mask. In addition, we used the lesion frequency map to exclude voxels, which was labelled as a lesion voxel in one or more patients, which was done to restrict the analysis to non-lesion brain tissue. For descriptive purposes, all group statistical parametric maps used an uncorrected, cluster-forming threshold of p < 0.001.

Clinical, behavioural and structural MRI measures are given as mean (± standard deviation) and were analysed with SPSS software (version 22, IBM Corp., Armonk, New York, USA), using repeated measures ANOVA, t-tests and Pearson or Spearman correlation when appropriate. Significance threshold was set at p < 0.05.

## Results

### Clinical and grip force data

Forty-nine of the 50 patients underwent MRI scanning, but three fMRI data sets could not be used because of motion artefacts or insufficient task compliance. Two additional patients were excluded because of high BDI-II scores. In these two patients, the total BDI-II score indicated the presence of severe symptoms of depression, although these patients had no clinical diagnosis of depression. Thus, 44 MS patients (27 FMS patients and 17 NFMS patients) and 25 healthy controls were included in the group analyses. Clinical characteristics and group data of the structural MRI analyses are summarized in Table 1. Healthy controls and the MS patients had comparable age and sex distributions. Patients had higher total, motor, and cognitive FSMC scores than healthy controls. Patients also had significantly higher average BDI-II, PQSI, and JTHFT scores than healthy controls.

**Table 1:** Clinical characteristics of MS patients and healthy controls.

		MS n = 44			HC n = 25		
* *	Mean	Range	SD	Mean	Range	SD	p
Age	35.9	(22–53)	8.8	35.8	(19–55)	10.6	.979
Gender _(M:F)_	14:30	32%:68%		9:16	36%:64%		.723
Median EDSS	2.5	(0–3.5)	1.0				
Disease duration	6.3	(0–28)	5.2				
Treatment	40 MS	90.9%					
**Clinical scores**							
FSMC _TOTAL_*	59.3	(20–92)	21.3	28.0	(20–46)	8.2	.000
FSMC _MOTOR_*	28.8	(10–45)	10.6	12.9	(10–23)	3.2	.000
FSMC _COGNITIVE_*	30.5	(10–48)	11.9	15.0	(10–28)	5.6	.000
BDI—II*	7.2	(0–22)	6.0	1.6	(0–11)	2.8	.000
PSQI*	5.2	(1–18)	3.7	3.4	(1–5)	1.4	.005
ESS	8.2	(2–17)	3.9	6.4	(0–14)	4.0	.080
PASAT	50.1	(33–60)	7.5	51.1	(43–59)	5.0	.506
SDMT	54.2	(35–89)	10.5	56.5	(41–70)	6.7	.280
JTHFT _RIGHT HAND_*	37.7	(30–53)	4.2	35.4	(29–41)	3.5	.026
9-HPT _RIGHT HAND_	15.9	(13–24)	2.0	15.7	(13–19)	1.8	.628
**Structural MRI metrics**							
Mean TIV	1561.3		141.4	1594.9		154.7	.362
Mean WM	482.7		59.9	500.2		56.9	.135
Mean GMV	637.5		47.9	657.2		47.9	.170
Mean WMHV	5.9	(0.3–30.7)	6.5				

* = p–value < 0.05

Abbreviations: Age = Age in years, BDI—II = Beck depression inventory version II, Disease duration = Years since diagnose, EDSS = Expanded disability status score, ESS = Epworth sleepiness scale, FMS = MS patients with fatigue, FSMC_COGNITIVE_ = FSMC cognitive score, FSMC_MOTOR_ = FSMC motor score, FSMC_TOTAL_ = Fatigue scale for motor and cognitive functions total score, Gender _(M : F)_ = Male: female ratio, HC = Healthy controls, GMV = Grey matter volume in millilitre, JTHFT = Jebsen-Taylor hand function test, MS = Multiple sclerosis, NFMS = MS patients without fatigue, WMHV = White matter hyperintensities volume (i.e. white matter lesions, in millilitre), 9-HPT = Nine hole peg test, p = P–value, PASAT = Paced auditory serial addition test, PSQI = Pittsburgh sleep quality index, SD = Standard deviation, SDMT = Symbol digit modalities test, TIV = Total intracranial (volume in millilitre), Treatment = In treatment with multiple sclerosis disease modifying drugs, WMV = White matter volume in millilitre.

In the patient group, individual FSMC_MOTOR_ scores showed a positive correlation with BDI-II scores (r = 0.53, p < 0.001) and EDSS scores (r = 0.47, p = 0.001). The individual FSMC_MOTOR_ scores did not correlate with disease duration or total white matter lesion volume. Accordingly, the FMS group had higher BDI-II and EDSS scores than the NFMS patients, while other clinical scores did not differ significantly between the two groups (Table 2). TIV and GMV were higher in the NFMS group than in the FMS group (Table 2).

**Table 2:** Clinical characteristics of the MS patients.

		NFMS n = 17			FMS n = 27		
	Mean	Range	SD	Mean	Range	SD	p
Age	34.5	(22–50)	8.3	36.7	(25–53)	9.7	.411
Gender _(M : F)_	8:9	47%:53%		6:21	22%:78%		.085
Median EDSS*	2.0	(0–3.5)	1.1	2.5	(0–3.5)	0.8	.023
Disease duration	6.4	(1–28)	6.4	6.2	(0–16)	4.5	.891
Treatment	15 MS	88.2%		25 MS	92.6%		.624
**Clinical scores**							
FSMC _TOTAL_*	38.3	(20–57)	14.3	72.5	(45–92)	12.5	.000
FSMC _MOTOR_*	17.4	(10–25)	5.5	35.9	(27–45)	5.4	.000
FSMC _COGNITIVE_*	20.9	(10–42)	9.6	36.6	(15–48)	8.9	.000
BDI—II*	3.4	(0–10)	3.3	9.7	(0–22)	6.2	.000
PSQI	4.4	(2–9)	1.7	5.7	(1–18)	4.5	.171
ESS	6.8	(2–17)	4.3	9.0	(3–17)	3.5	.062
PASAT	52.2	(41–60)	5.3	48.8	(33–60)	8.4	.102
SDMT	55.4	(40–89)	12.3	53.5	(35–71)	9.3	.557
JTHFT _RIGHT HAND_	37.3	(13–22)	2.4	37.9	(13–24)	5.1	.648
9-HPT _RIGHT HAND_	16.0	(32–40)	2.0	15.9	(30–53)	2.1	.954
**Structural MRI metrics**							
Mean TIV*	1620.4		147.4	1524		126.3	.026
Mean WM	496.7		69.2	473.8		52.7	.262
Mean GMV*	656.2		42.8	625.7		47.9	.038
Mean WMHV	6.9	(0.5–30.7)	7.8	5.3	(0.3–22.9)	5.5	.420

* = p–value < 0.05

Abbreviations: Age = Age in years, BDI—II = Beck depression inventory version II, Disease duration = Years since diagnose, EDSS = Expanded disability status score, ESS = Epworth sleepiness scale, FMS = MS patients with fatigue, FSMC_COGNITIVE_ = FSMC cognitive score, FSMC_MOTOR_ = FSMC motor score, FSMC_TOTAL_ = Fatigue scale for motor and cognitive functions total score, Gender _(M : F)_ = Male: female ratio, HC = Healthy controls, GMV = Grey matter volume in millilitre, JTHFT = Jebsen-Taylor hand function test, MS = Multiple sclerosis, NFMS = MS patients without fatigue, WMHV = White matter hyperintensities volume (i.e. white matter lesions, in millilitre), 9-HPT = Nine hole peg test, p = P–value, PASAT = Paced auditory serial addition test, PSQI = Pittsburgh sleep quality index, SD = Standard deviation, SDMT = Symbol digit modalities test, TIV = Total intracranial (volume in millilitre), Treatment = In treatment with multiple sclerosis disease modifying drugs, WMV = White matter volume in millilitre.

Patients performed the precision grip task equally well as healthy controls during fMRI. Mean grip force was 16.17 (±4.67) N in healthy controls and 16.20 (±2.40) N in MS patients. The mean variability across subjects of the applied force was 0.54 (±0.80) N in healthy controls and 0.52 (±0.31) N in patients. Repeated-measures ANOVA showed no time or group effect and no interaction between time and group for mean force levels and the variability or grip force during the task.

### Task-related activity during tonic grip force task

#### Task-related activation

**Fig 3:**
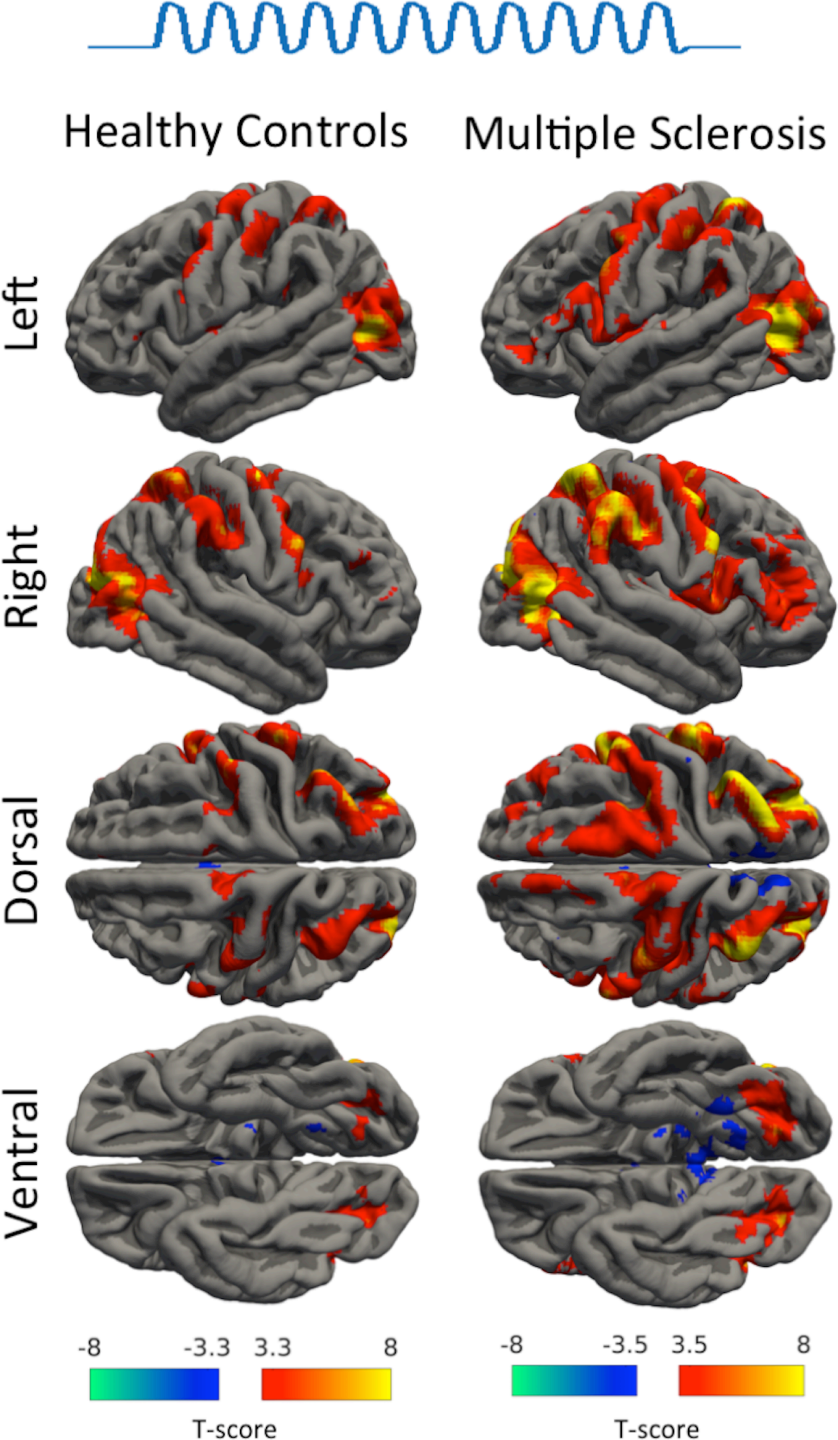
Main effect of the precision grip task. T-score maps showing the brain activation during the tonic precision grip task in healthy controls and patients with MS. For visualisation purposes, the maps were thresholded at an uncorrected p-value of < 0.001.

The tonic grip force task activated a well-known sensorimotor network that has been shown to be engaged in visually guided control of precision grip force [44]. The network comprised cortical clusters in the prefrontal, premotor, sensorimotor, parietal and occipital cortex, as well as cerebellum and basal ganglia bilaterally. The task-related activity pattern was very similar in both groups with no significant between-group differences in task-related activation between MS and healthy controls (Fig 3) or between the FMS and NFMS groups.

#### Relation between task-related activation and motor fatigue

**Fig 4:**
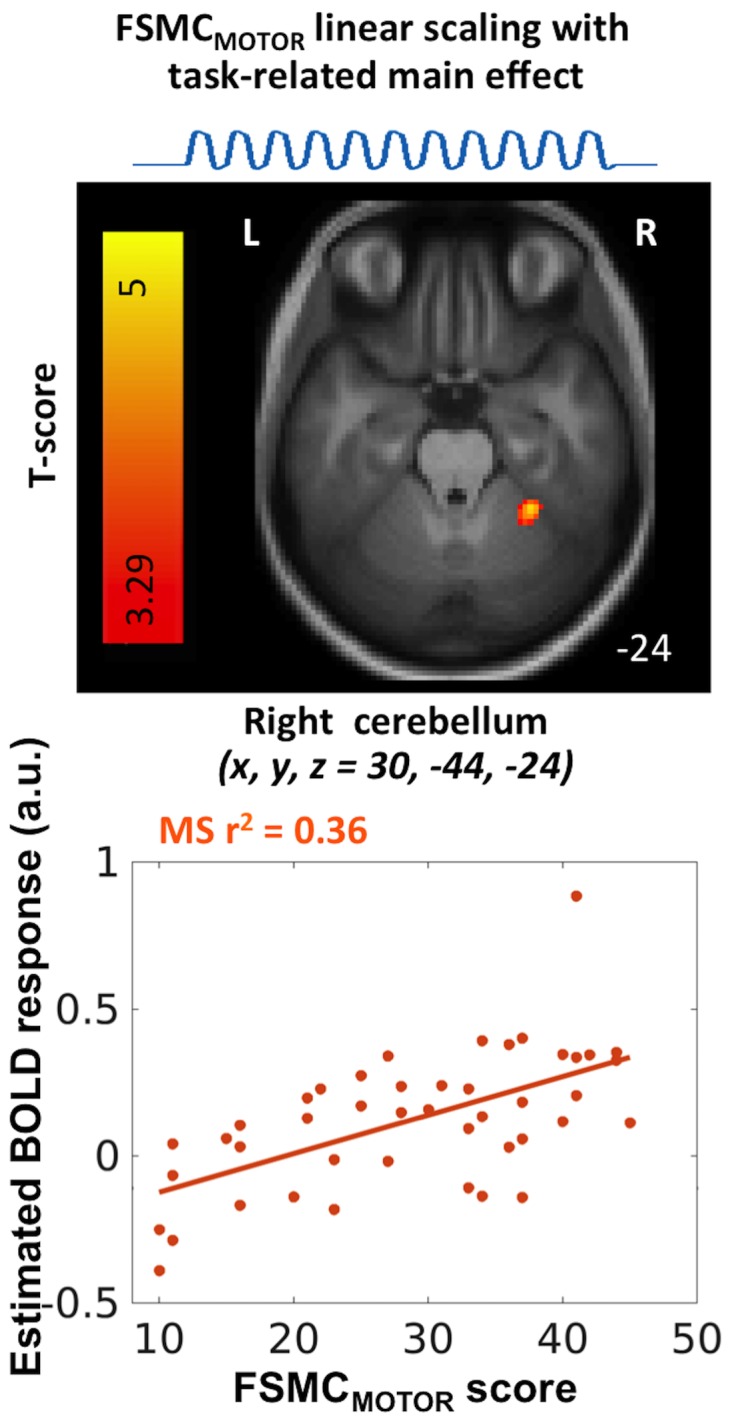
MS patients—within group analysis. Linear scaling of the constant task-related activation during a non-fatiguing grip force task, with the amount of experienced fatigue during daily life, as indexed by the FSMC_MOTOR_ score. In right motor cerebellum there was a linear increase in task-related activation in the MS group with increasing motor fatigue (p_SVC_ = 0.046, r^2^ = 0.36).

Patients with MS showed a linear relationship between task-related activation and individual FSMC_MOTOR_ scores in the right upper cerebellar lobule VI (Table 3). The cerebellar cluster was located in the hand motor representation ipsilateral to the hand performing the task (Fig 4), indicating that cerebellar task-related activity scaled positively with the magnitude of self-reported motor fatigue and fatigability during everyday life. No linear relationship between individual fatigue scores and task-related cerebellar activity was found in healthy controls.

**Table 3:** Group results of the fMRI data.

			Peak	MNI- coordinates			P value	Cluster size
Contrast	Region	Side	T value	x	y	z		
**Main effect of task—Linear scaling of the main effect of task with FSMC**_**MOTOR**_ **scores**								
**MS** *(positive)*	Cerebellum VI	R	4.68	30	-44	-24	0.046 (SVC)	35
**HC > MS**	STG	R	5.98	48	-34	20	0.004	123
**NFMS > FMS**	PMd	L	6.27	-30	4	46	0.017	87
	PMd	L	4.75	-26	-12	74	0.049 (SVC)	39
	dmPFC	L	5.96	-12	38	54	0.038	70
**First-order time modulation of task-related activity—Linear change in task-related activity**								
**MS** *(negative)*	Cerebellum VI	L	4.75	-34	-46	-32	0.042 (SVC)	30
**HC > MS**	PCC	R/L	5.62	2	-44	10	0.018	685
	Lingual gyrus							
**First-order time modulation of activity—Linear scaling the with FSMC**_**MOTOR**_ **scores**								
**HC > MS**	PMd	R	4.60	22	-12	78	0.031 (SVC)	31
	Cerebellum	L	4.57	-16	-58	-12	0.034 (SVC)	11
	Lingual gyrus	L	7.17	-16	-54	-10	0.000	185

**Group results of the fMRI data.** T-values and p-values refer to the voxel showing peak difference in a given cluster. Cluster extent is defined by an uncorrected cluster-forming extent threshold of p < 0.001. The p-values reflect significant activity changes at peak-voxel level (p-value < 0.05) after whole-brain FWE correction for multiple comparisons. SVC = small volume correction: For voxels within the a priori defined ROIs, FWE correction only considered the voxels within the mask comprising all pre-defined ROIs. As for the whole-brain analysis, the FWE method was applied at the peak-voxel level. Only voxels with a FWE corrected p-value < 0.05 were considered to be significant. Cerebellum VI = Cerebellum lobe VI. dmPFC = Dorsomedial prefrontal cortex. FMS = Fatigued MS patients. FSMC_MOTOR_ = Fatigue Scale for Motor and Cognitive Functions, motor subscale. HC = Healthy controls. MS = Multiple sclerosis. NFMS = non-fatigued MS patients. PCC = Posterior cingulate cortex. PMd = Dorsal premotor cortex. STG = Superior temporal gyrus.

**Fig 5:**
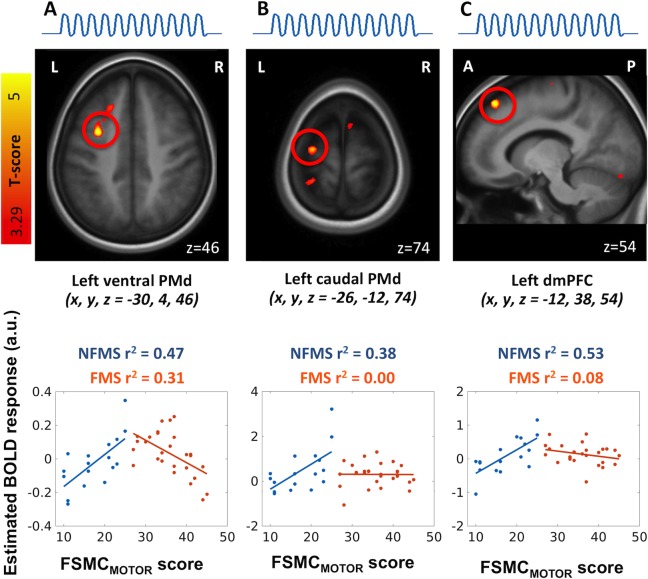
The non-fatigued MS patients compared to the fatigued MS patients. Linear scaling of the constant task-related activation during a non-fatiguing grip force task, with the amount of experienced fatigue during daily life, as indexed by the FSMC_MOTOR_ scores in the non-fatigued MS patients compared to the fatigued MS patents.**(A**) The left ventral part of the dorsal premotor cortex (PMd) (p_FWE_ = 0.017), **(B)** left caudal part of the dorsal premotor cortex (PMd) (p_SVC_ = 0.049) and **(C)** left dorsomedial prefrontal cortex (dMPFC) showed increased linear correlation between task-related activation and FSMC_MOTOR_ scores in the non-fatigued MS patients (blue) compared with the fatigued MS patients (red) (p_FWE_ = 0.038).

We divided the patient group in patients with fatigue (FMS group) and without fatigue (NFMS group). When comparing these groups, we found regional differences in the scaling between task-related activity and FSMC_MOTOR_ scores in two clusters within the left PMd, peaking at MNI-coordinates x, y, z = -30, 4, 46 and x, y, z = -26, -12, 74 (Table 3). In these PMd regions, NFMS patients who did not experience excessive motor fatigue during everyday life showed a linear increase in task-related activation with the magnitude of experienced motor fatigue (Fig 5). This relationship between task-related activity and FSMC_MOTOR_ scores was absent in FMS patients who reported excessive levels of motor fatigue (Fig 5). The same pattern was found in a cluster in the dorsomedial prefrontal cortex (dmPFC) rostral to the pre-supplementary area. Here task-related activity scaled positively with individual FSMC_MOTOR_ scores in the NFMS group, but not in the FMS group (Fig 5).

#### Linear changes in activation during repeated task performance

Compared to patients with MS, healthy controls showed a stronger time-dependent increase in task-related activity in the right posterior cingulate cortex and adjacent lingual gyrus (Table 3). Conversely, patients showed no brain region where task-related activation increased more strongly with time than in healthy controls. We also found no differences in time-dependent task modulation between the FMS and NFMS groups.

Considering only the patients with MS, we found a negative linear effect of time on task-related activity in the motor territory of the left cerebellum contralateral to the hand performing the grip force task (Table 3). In contrast, no brain region showed a significant linear increase or decrease in task-related activity with the duration of task performance in healthy controls.

**Fig 6:**
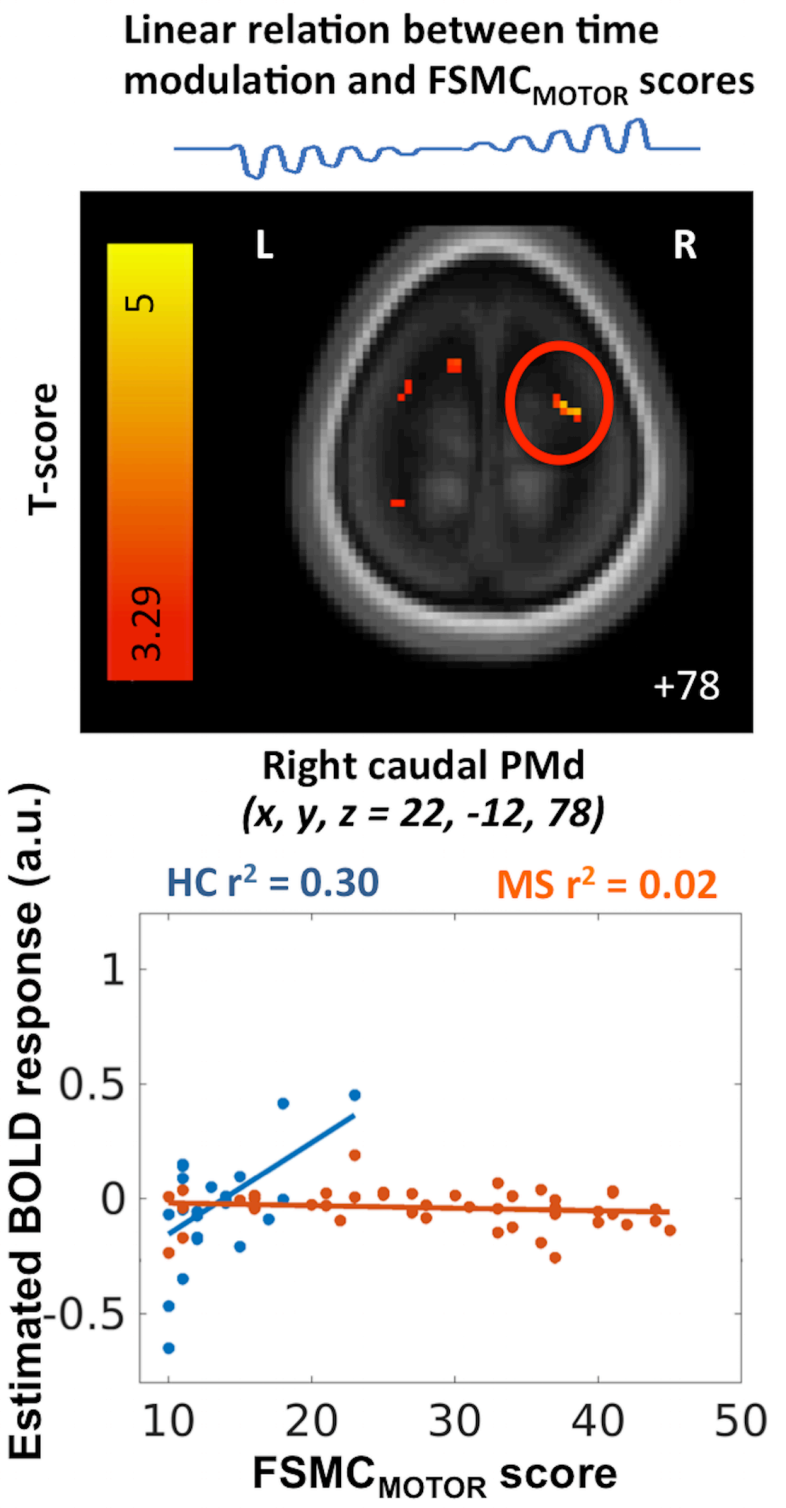
MS patients compared to healthy controls. MS patients’ task-related activity scaled with individual FSMC_MOTOR_ scores relatively to the scaling in healthy controls. Healthy controls showed a stronger time-dependent increase in task-related activity in the right dorsal premotor cortex (PMd) than patients with MS (p_SVC_ = 0.031). In healthy controls, the linear time modulation of task scaled with the individual FSMC_MOTOR_ scores (blue). The more controls experienced fatigue during daily life; the more they displayed a linear increase in task-related activity with time during the non-fatiguing grip force task. This relationship was not present in MS patients (red).

A correlation analysis between the individual FSMC_MOTOR_ scores and the task-related brain activity revealed a difference in the linear time-dependent modulation of task-related activity (Fig 6). In right caudal PMd, the time-dependent increase in task-related activity scaled more strongly with the individual FSMC_MOTOR_ scores in healthy controls than in patients with MS (Table 3). The higher the individual FSMC_MOTOR_ scores, the stronger the time-dependent increase of task-related PMd activity in healthy controls, but not in MS patients (Fig 6). The same pattern was found in a rostromedial cluster of the left cerebellum and lingual gyrus. When each group was tested separately, the relationship between time-dependent modulation of PMd activity and FSMC_MOTOR_ scores did not reach significance.

There were no clusters in the brain where the time-dependent increase in task-related activity correlated more strongly with FSMC_MOTOR_ scores in MS patients than in controls. There was also no influence of the FSMC_MOTOR_ scores on time-dependent activity changes, when contrasting FMS and NFMS patients.

## Discussion

Here we used task-related fMRI to map sensorimotor brain activity evoked by a tonic right-hand grip force task in mildly impaired patients with MS and healthy controls. We identified distinct regions in the cerebellum and premotor cortex where sensorimotor activity in the non-fatigued state scaled with the amount of motor fatigue that patients experienced during everyday life. Task-related activity in right motor cerebellum increased in proportion with self-reported fatigue. Furthermore, patients lacked a “normal” upscaling of regional task-related activity in premotor and dorsomedial prefrontal areas with the level of subjectively experienced fatigue. As pointed out above, self-reported “motor fatigue”, as probed with the FSMC_MOTOR_ or other standardized clinical scales, reflects both effort-independent “trait” fatigue as well as effort-induced “state” fatigue (i.e., fatigability) [4–6].

### Cerebellar activity and self-experienced fatigue

The right motor cerebellum ipsilateral to the grasping hand was the only brain region, where functional activation during a non-fatiguing tonic grip force task scaled significantly with the amount of experienced motor fatigue in patients with MS (Fig 4). This finding confirms and extends previous fMRI studies on fatigue in MS. In 14 mildly affected patients with relapsing-remitting MS, subjective fatigue during everyday life correlated positively with task-related activation of the right cerebellar lobule VI ipsilateral to the moving hand [9]. In that study, patients had to generate externally paced finger-to-thumb opposition sequences with their right hand at a rate of 2 Hz during fMRI. A possible correlate at the metabolic level has also been reported in a study using 18F-fluorodeoxyglucose positron emission tomography [45]. Likewise, an early fMRI study reported a stronger overall activation of the right motor cerebellum in mildly impaired MS patients suffering from fatigue as opposed to those without fatigue, when patients performed flexion–extension finger movements with their right hand at a paced rate of 1 Hz [10]. In summary, our and previous fMRI studies consistently show across a range of non-fatiguing manual tasks, that task-related activation of the sensorimotor cerebellum reflects how much fatigue patients experience during their everyday live.

Functional and structural changes in the cerebellum contribute significantly to disease-induced motor disability in MS [46, 47] as well as to cognitive and emotional disturbances [48]. The motor cerebellum is involved in the detection of motor errors and their correction during on-going movements and motor learning [49, 50] and secures temporal and spatial precision and fluency of movements based on internal models [51, 52]. Accordingly, in addition to individual fatigue scores, the temporal accuracy of repetitive finger movements correlated positively with the activity in right cerebellar lobule VI and temporal accuracy during the task correlated positively with the self-reported fatigue in the study by Pardini et al. [9]. This ‘‘fatigue-motor performance paradox” prompted Pardini et al. to propose that patients who experience fatigue may be the ones who excessively monitor their errors to optimize performance [9]. In other words, scaling-up cerebellar sensorimotor control might be a compensatory mechanism to secure good performance but at the same time cause fatigue. Excessive sensorimotor processing in the cerebellum may lead to a faster exhaustion of neural resources, and thereby promote effort-induced experience of fatigue. Given that the cerebellum plays an important role in a range of non-motor functions [53], a similar consideration may apply for non-motor aspects of fatigue and altered processing in other non-motor territories in the cerebellum.

The increase in cerebellar motor activity with increasing individual fatigue scores was found during both tonic motor activity in the present study and phasic repetitive activity in the study by Pardini et al. [9]. The sensorimotor task employed by Pardini et al. [9] required temporal error processing to optimize the timing of finger movements relative to an auditory pacing cue. In contrast, the sensorimotor task used in the present study engaged the processing of magnitude errors in force output based on the simultaneous visual display of the exerted force and the target force. Although the two tasks implicated different types of error processing, they both required the continuous integration of external target signals with sensory feedback created by the motor output. Both sensorimotor settings revealed a positive relationship between task-related motor activation of ipsilateral cerebellar lobule VI and self-reported magnitude of fatigue, supporting the notion that the motor cerebellum excessively monitors performance during relatively simple motor tasks that has yet not induced fatigability.

In the left sensorimotor cerebellum contralateral to the grasping hand, patients displayed a time-dependent linear decrease in task-related activity during the fMRI session (Fig 4). In addition, the time-dependent linear increase in task-related activity in the rostromedial part of the left motor cerebellum scaled more strongly with the individual FSMC_MOTOR_ scores in healthy controls than in patients with MS. The higher the individual FSMC_MOTOR_ scores, the stronger was the time-dependent increase of task-related cerebellum activity during the fMRI session in healthy controls, but not in MS patients. This finding indicates that not only the constant level of ipsilateral cerebellar sensorimotor activation, but also the lack of temporal modulation of contralateral cerebellar activity during continued task performance may be related to motor fatigue experienced during everyday life.

### Premotor activity and self-experienced fatigue

The premotor cortex forms multiple reciprocal loops with the parietal cortex, through which sensory information is processed and transformed into actions [54–56]. The PMd is a key region for manual motor control and is involved in response selection and non-routine visuo–motor mapping [57–59]. PMd participates both in motor planning and execution [44], displaying a rostro-caudal functional gradient with the more caudal part being associated more closely with motor execution [58, 60, 61].

In accordance with our hypothesis, several clusters in PMd showed an altered activation profile in patients with MS that change in proportion with the amount of motor fatigue experienced during daily life. In the caudal part of right PMd, patients and controls showed differences in the linear time-dependent modulation of task-related activity during the fMRI session, which scaled linearly with the individual FSMC_MOTOR_ scores. In the right caudal PMd, healthy controls showed a stronger time-dependent increase of task-related activity with the experienced magnitude of motor fatigue than MS patients (Fig 6). This difference between groups suggests that healthy individuals gradually increase task-related recruitment of the PMd during repeated task blocks, the more they experience fatigue during daily life. Of note, none of the healthy controls reported a level of fatigue that was of clinical relevance. Hence, the relationship found in healthy controls between the time-dependent modulation of PMd activity and the FSMC_MOTOR_ scores applies to normal inter-individual variations in the physiological range. One might speculate that the ability to recruit the right PMd during continuous task performance might protect against the occurrence of excessive fatigue or fatigability.

The positive relationship between the temporal modulation of PMd activity and experienced daily-life fatigue was absent in the patient group, even though patients showed a wider inter-individual spread and overall higher FSMC_MOTOR_ scores. Using a non-fatiguing hand flexion-extension task, a recent fMRI study found that MS patients suffering from fatigue showed a reduced activation change over time in left putamen and precentral gyrus compared to healthy controls [8]. Our results extend the findings showing a deficient time-dependent task-related activation over time in the right caudal PMd the more patients experienced motor fatigue during daily life. In addition, the comparison of the FMS and NFMS groups revealed that overall task-related activity in a ventral cluster and a caudal cluster of left PMd depended on the presence of motor fatigue during daily life (Fig 5A and 5B). In these left-hemispheric clusters, task-related activity scaled differentially with the magnitude of fatigue in the FMS groups relative to the NFMS group. NFMS patients, who had FSMC_MOTOR_ scores close to the normal range, showed a linear increase in task-related activation of left PMd with their individual FSMC_MOTOR_ scores. In contrast, FMS patients, who had abnormally high FSMC_MOTOR_ scores, showed a linear decrease or no change in task-related activation depending on their individual FSMC_MOTOR_ scores. In agreement with our findings, Specogna et al. [13] found stronger task-related activity in a ventral cluster of the left PMd during a self-paced sequential finger-tapping task in NFMS patients relative to healthy controls. This increase in task-related premotor activation was not present in MS patients with fatigue [13]. The results suggest a link between the inability of task-related PMd recruitment and the daily experience of motor fatigue in patients with MS. The NFMS group upscaled task-related activation of PMd, the more patients experienced signs of motor fatigue during everyday life. Conversely, the FMS group failed to up-scaled the task-related activation of PMd with the increasing severity of daily-life motor fatigue. Based on these findings, we hypothesize that patients who suffer from motor fatigue may fail to gradually upscale task-related engagement of PMd. The inability to sufficiently recruit PMd during prolonged performance of non-fatiguing motor tasks might reflect deficient sensorimotor integration within the PMd and contribute to abnormal fatigability during daily activities.

### No consistent abnormality of task-related activity of the putamen

The putamen was the only pre-defined region of interest where we found no significant alteration of the regional activation profile in relation to fatigue. This negative finding may be related to the nature of the task, which did not rely critically on the basal ganglia, but rather on cerebellar integration of the produced motor output and the visual and somatosensory input to produce a constant force output. Other motor tasks, for instance tasks that require repetitive or sequential movements, might reveal altered activity patterns in the basal ganglia that are related to motor fatigue in MS as seen in other studies [8, 14].

### Motivational drive and dorsomedial prefrontal cortex

The left dorsomedial part of the prefrontal cortex showed a difference in task-related activation in the patient group depending on whether patients suffered from excessive fatigue or not. Patients without fatigue showed a positive relationship between task-related dmPFC activity and their individual FSMC_MOTOR_ scores, whereas patients with motor fatigue did not show this pattern (Fig 5C). In the FMS group, dmPFC activity did not change in proportion with the magnitude of self-reported motor fatigue. In accordance with our finding, a recent task-related fMRI study reported reduced activation of dmPFC in FMS patients compared to NFMS patients and HC during a repetitive extension-flexion task [8].

Furthermore, a fMRI study in healthy individuals has identified a motivational action control circuit that secures consistent force production “despite changes in emotional context” and includes the dmPFC, PMv and PMd [62]. The study showed that dmPFC and PMv increase task-related activity during a visually cued phasic grip task, when the task context is emotionally salient [62]. Additional connectivity analysis revealed a stronger functional coupling of dmPFC with ventral and dorsal portions of premotor cortex in an emotionally salient versus a neutral context. In present study, we find that the more severe motor fatigue the patients are experiencing, the more the task-related activity was downscaled in left dmPFC and PMd. Taking into account the work by Coombes et al. (2012) in healthy individuals, we speculate that a downscaling of dmPFC and PMd activity may point to a dysfunction of the motivational action control circuit in MS patients who suffer from motor fatigue, reducing the internal motivational drive.

### Methodological considerations and limitations of this study

The FSMC questionnaire has been developed to assess fatigue in MS and requests the subject to evaluate how much they experience an extreme form of tiredness (fatigue) i.e. the “overwhelming state of lethargy, exhaustion and lack of energy which comes on abruptly and is unrelated to any obvious external causes” during their day-to-day life [22]. Hence, the questionnaire probes the subjective experience of fatigue during day-to-day life in general rather than the momentary expression of fatigue at the time of examination. The questionnaire contains items that capture effort-independent “trait” fatigue as well as effort-related “state” fatigue (i.e., fatigability). Since we used the FSMC_MOTOR_ score as proxy for motor fatigue in this study, it is impossible to disentangle whether the brain activation changes are related to “trait” fatigue, fatigability and/or an interaction between the two. Furthermore, it remains controversial whether”trait” fatigue and “state” fatigability can be dissociated [63]. While fMRI can help to pinpoint abnormal patterns of regional brain activations related to motor fatigue in MS, fMRI provides no insights into the specific neurobiological mechanisms through which MS gives rise to these functional activation changes [64]. Furthermore, whether these brain activation changes are a response, cause or mediator of fatigue cannot be inferred from the present study. This question may be tackled with an interventional study design in which the amount of fatigue or the activation patterns are altered by a targeted intervention (e.g. pharmacological therapy or focal brain stimulation).

The present study deliberately focussed on relatively mildly affected patients with a relapsing-remitting presentation of MS. Therefore the generalizability to other clinical forms of MS such as primary or secondary progressive MS or more severely impaired patient groups is limited.

We used a visual-spatial grip force control task to study neural correlates of motor fatigue in sensorimotor brain networks. While the focus was on the motor component of fatigue, the motor and cognitive subscales of the FSMC questionnaire were highly correlated in our sample, in agreement with the literature [23]. Therefore, it remains unclear how much the present findings are really specific to motor aspects of fatigue or generalize to the cognitive domain.

In addition, the task did not activate motor networks implicated in the control of other motor effectors such as legs (gait) or mouth (speech and swallowing). The relatively simple task also did not capture the physical and mental challenges of skilled manipulative actions during everyday life. This implies that the present findings need to be interpreted in the context of the specific motor task and generalization to other motor tasks should be made with caution. Another inherent limitation is that MS leads to motor impairment, and the effects of motor impairment on task-related activation may confound the activation patterns related to motor fatigue. However, we consider it highly unlikely that the present findings were accounted for by disease-related impairment of hand function. Inter-individual differences in hand function were taken into account in our statistical model. In contrast to task-related fMRI, resting state fMRI has the advantage of not being confounded be type of task type or task performance.

Patients and healthy controls performed the grip force task equally well during the fMRI session, and there were no between-group differences in task-related brain activation. However, the results of this study are limited by the lack of controlling for regional lesion burden.

Finally, it is generally accepted that fatigue and depression constitute two different but closely related conditions [65]. This results in an inherent problem when studying fatigue, as patients rating high on a fatigue questionnaire will also score high on a depression rating scale, even when excluding patients with a clinical diagnosis of major depression. In this study, to make sure the differences in brain activation was not biased by depression none of the patients had a clinical diagnosis of depression, patients with high BDI scores indicative of severe depressive symptoms were excluded and the comparison between the NFMS and FMS group was corrected for depression.

## Conclusions

The present study advances the current understanding of the neural underpinnings of fatigue in MS. We show that the functional activation of the motor cerebellum during a non-fatiguing tonic grip force task reflects the severity of motor fatigue and fatigability experienced during daily life. In dorsal and ventral premotor as well as dorsomedial prefrontal areas, inter-individual difference in task-related activation scaled with the level of experienced fatigue during everyday life. MS patients showed a reduced upscaling of task-related activity in this prefrontal-premotor network, the more the patients were affected by motor fatigue. In summary, the results are compatible with the notion that motor fatigue in MS is associated with an upscaling of cerebellar sensorimotor integration along with a downscaling of motivational drive and sensorimotor processing in prefrontal and premotor areas. However, whether these brain activation changes are a response, a cause or a mediator of fatigue cannot be concluded.
